# Sonographic evaluation of the degree of medial meniscal extrusion during Thessaly test in healthy knees

**DOI:** 10.1186/s12998-021-00390-5

**Published:** 2021-08-17

**Authors:** John C. Cho, Lauren Tollefson, Kenneth Reckelhoff

**Affiliations:** 1 Department of Clinical Sciences, Parker University, 2540 Walnut Hill Lane, Dallas, TX 75229 USA; 2 Chiropractic Department, D’Youville College, Buffalo, NY USA; 3 College of Chiropractic, Cleveland University, Kansas City, USA

**Keywords:** Medial meniscus, Ultrasonography, Thessaly test

## Abstract

**Objective:**

The Thessaly test is a commonly used orthopedic test for meniscus tear evaluation. The study’s objective is to evaluate the degree of medial meniscal extrusion during different loading phases of the Thessaly test.

**Methods:**

A convenience sample of 60 healthy knees (35 participants) was examined and the data sets were collected from October 8, 2018 through February 8, 2019. Sonographic measurement of the degree of physiologic extrusion of the medial meniscus deep to the medial collateral ligament was taken by two examiners at six different loading phases: supine, standing, 5° knee-flexion with internal (IR)/external (ER) rotation and 20° knee-flexion with IR/ER. The difference in meniscal extrusion by knee position was compared with ANOVA. Interexaminer reproducibility assessment was analyzed using limits of agreement.

**Results:**

The mean meniscal extrusion for each position was—supine: 2.3 ± 0.5 mm, standing: 2.8 ± 0.8 mm, 5° IR: 2.3 ± 0.9 mm, 5° ER: 2.4 ± 0.7 mm, 20° IR: 1.9 ± 0.8 mm, and 20° ER: 2.3 ± 0.7 mm. Significant increase in extrusion was observed from supine to standing (*p* < 0.05) and from 20° IR to 20° ER (*p* = 0.015). Significant decreased measurement was observed from standing to 5° IR (*p* < 0.05), 5° ER (*p* < 0.05), 20° IR (*p* < 0.05) and 20° ER (*p* < 0.05). There is no significant change between 5° IR and 5° ER (*p* = 1.0). Agreement parameters revealed that the differences between examiner measurements were minimal; 75% of both examiners’ meniscal extrusion measurements were within 1.0 mm with 97% of measurements falling within 2.0 mm.

**Conclusion:**

Our study’s novel findings showed various degrees of physiological extrusion of the medial meniscus in asymptomatic knees during the loading phases involved in the Thessaly test. Physiological MME does exist and should not be defaulted to pathologic meniscus as previously described. Agreement parameters suggest that measurement of meniscal extrusion during the Thessaly test is reproducible between different examiners.

## Background

Meniscal injury is common, with the incidence of injury resulting in meniscectomy of 61 per 100,000 persons [1]. Meniscal extrusion is described as external meniscal displacement and has been regarded as an indirect sign of meniscal injuries such as meniscal tear, meniscal maceration, cartilage damage, and knee malalignment; however, the exact cause of extrusion is not well understood [2–4]. Medial meniscal extrusion (MME) of > 2 mm [5] and > 3 mm [4] have been proposed as significant findings with associated meniscal injuries and/or osteoarthrosis. Although various meniscal injuries are known inducers of MME, the degree of normal, or physiological MME in healthy knees, has only been recently investigated [6].

For diagnosing intra-articular knee lesions, MR imaging is the gold standard after arthroscopic knee surgery [7]. When assessing morphologic changes of the meniscus, dynamic capability of sonographic evaluation allows visual assessment of the meniscus while exerting different loading stresses across the knee. Compared to MR imaging as the reference standard, reliability of sonographic assessment for MME seems promising [8].

The Thessaly test is a clinical test for detecting meniscal tear, first described in 2005 at the University of Thessaly in Greece [9]. Since its inception, numerous studies investigated diagnostic ability of the Thessaly test. For instance, Harrison et al. examined 116 patients undergoing knee arthroscopic examination for suspected meniscal tear where the Thessaly test demonstrated 90.3% sensitivity, 97.7% specificity, and 88.8% diagnostic accuracy [10]. In another study of 86 patients comparing diagnostic accuracy of clinical examination (using McMurray’s, Apley’s and Thessaly’s test at 20° flexion) versus MRI and the gold standard arthroscopy, MRI demonstrated the highest diagnostic power, followed by the Thessaly test [11].

Contrarily, there are studies which repudiate these results. In a prospective diagnostic accuracy study of a cluster of physical examination tests (Thessaly’s, Apley’s, McMurray’s and joint line tenderness) versus MRI in 292 patients with knee pathology, the Thessaly test demonstrated only 59% diagnostic accuracy [12]. Similar conclusions derived from other authors have challenged the diagnostic accuracy as too low to be considered as routine clinical value [13, 14].

The Thessaly test is performed by moving the femur through internal and external rotation on the tibia in different knee angles (5° and 20° of flexion) as a means of placing the meniscus under dynamic stress [9]. The amount of load applied to the medial meniscus is indirectly assessed under ultrasound through measuring the degree of MME. We hypothesized that if the Thessaly test stresses the knee joint, increase in MME would be observed. It is also hypothesized that the largest degree of MME will be noted at 20° of knee flexion. To the best of our knowledge, this is the first study investigating sonographic characteristics of the medial meniscus under various loading phases of the Thessaly test.

## Methods

This was a repeated measures design with students and employees at our institution as a convenience sample. Signed informed consent was obtained from all participants and the data sets were collected from October 8, 2018 through February 8, 2019. Ethical approval was received by our Institutional Review Board (IRB Protocol# A- 00181).

The inclusion criterion was to have asymptomatic knees without any of the following factors: (1) history of knee surgery, (2) known osteoarthritis of the knee and/or (3) injury to the knee that resulted in physician consultation for evaluation and/or therapy. Exclusion criterion was a positive orthopedic examination (i.e., McMurray’s test, Anterior and posterior drawer test).

**Fig. 1:**
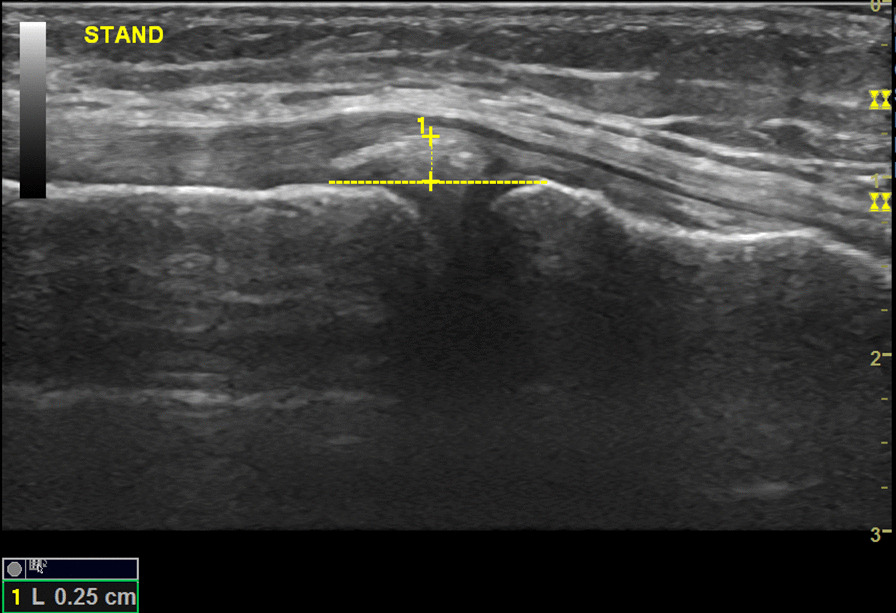
Sonography of the medial meniscus deep to the MCL while standing

Diagnostic ultrasound (Mindray M5; Shenzhen, China) using a 7–12 MHz linear array transducer was used to measure degree of physiological MME (in millimeter) of the medial meniscus at six different loading phases: (1) supine, (2) standing, (3) 5° knee flexion with internal rotation (IR), (4) 5° knee flexion with external rotation (ER), (5) 20° knee flexion with IR and (6) at 20° flexion with ER. The medial meniscus was scanned with the transducer parallel to the medial collateral ligament (MCL) to assure consistency between the examiners and the loading phases. This site of measurement was previously used by other investigators [6, 8]. For the measurement, an initial line was drawn connecting the free edges of the medial tibial cortex to the medial femoral cortex. A second line was drawn tangential to the apex of the medial meniscus from which a measurement was drawn to the initial line for MME (Fig. 1). All images were saved on the ultrasound unit (without the measurements). For the interexaminer reproducibility assessment, each examiner, JC and LT (10 years and 1 year experience in musculoskeletal ultrasound, respectively) acquired their own set of images for measurements. Each loading phase was measured 3 times then averaged. A digital goniometer was used to bring the knee into 5° and 20° flexion.

Statistical analyses were conducted using the IBM SPSS Statistics 19 software (Chicago, IL). Interrater reproducibility assessment was analyzed with 95% limits of agreement as described by Bland–Altman [15]. A univariate one-way repeated measures ANOVA with pairwise comparisons was used to analyze the effect of knee position on MME. To test the assumption of normality, the Kolmogorov–Smirnov test was used and revealed a deviation from normality (*p* = 0.48) for the 5° ER position. The histogram of the dependent variable in the five ER position appeared symmetrical; thus, the violation of normality was deemed minor and within tolerance for parametric testing. Boxplot assessment of the dependent variable according to the levels of the independent variable revealed outliers present in the five ER and twenty ER positions. They were determined to not have a significant effect on the result and thus were left in place. No extreme values were detected. No data were missing in the analyses. Post-hoc power analysis was carried out using G*Power 3.1.9.2 software.

## Results

A total of 60 healthy knees were scanned (35 subjects; mean age = 29; age range = 21–39); there were no exclusions for a positive orthopedic examination. Post-hoc power analysis with an effect size f(V) of 0.51 (determined according to partial η^2^ = 0.205), α of 0.05, and with the sample size of 60 revealed 81% power.

**Fig. 2:**
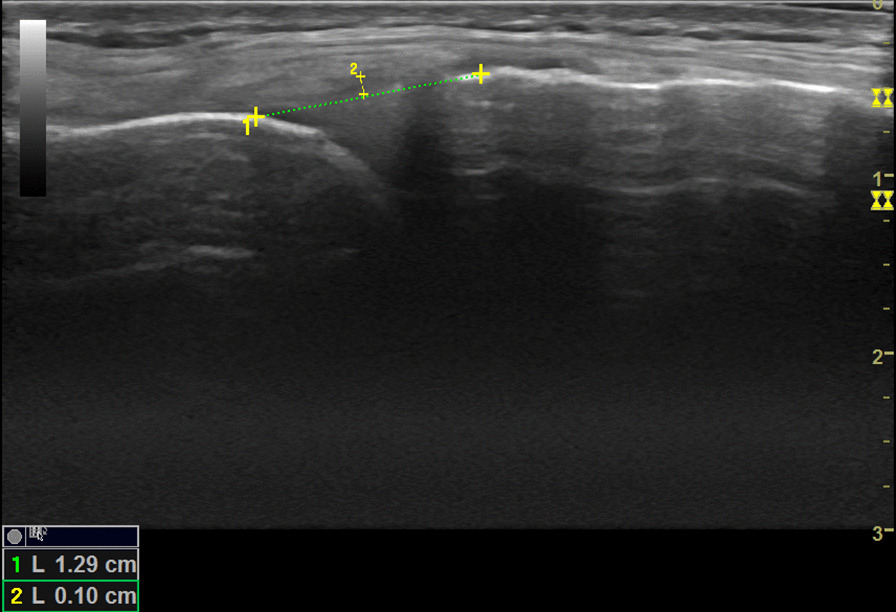
Sonography of the medial meniscus while knee in 20° flexion with internal rotation

**Fig. 3:**
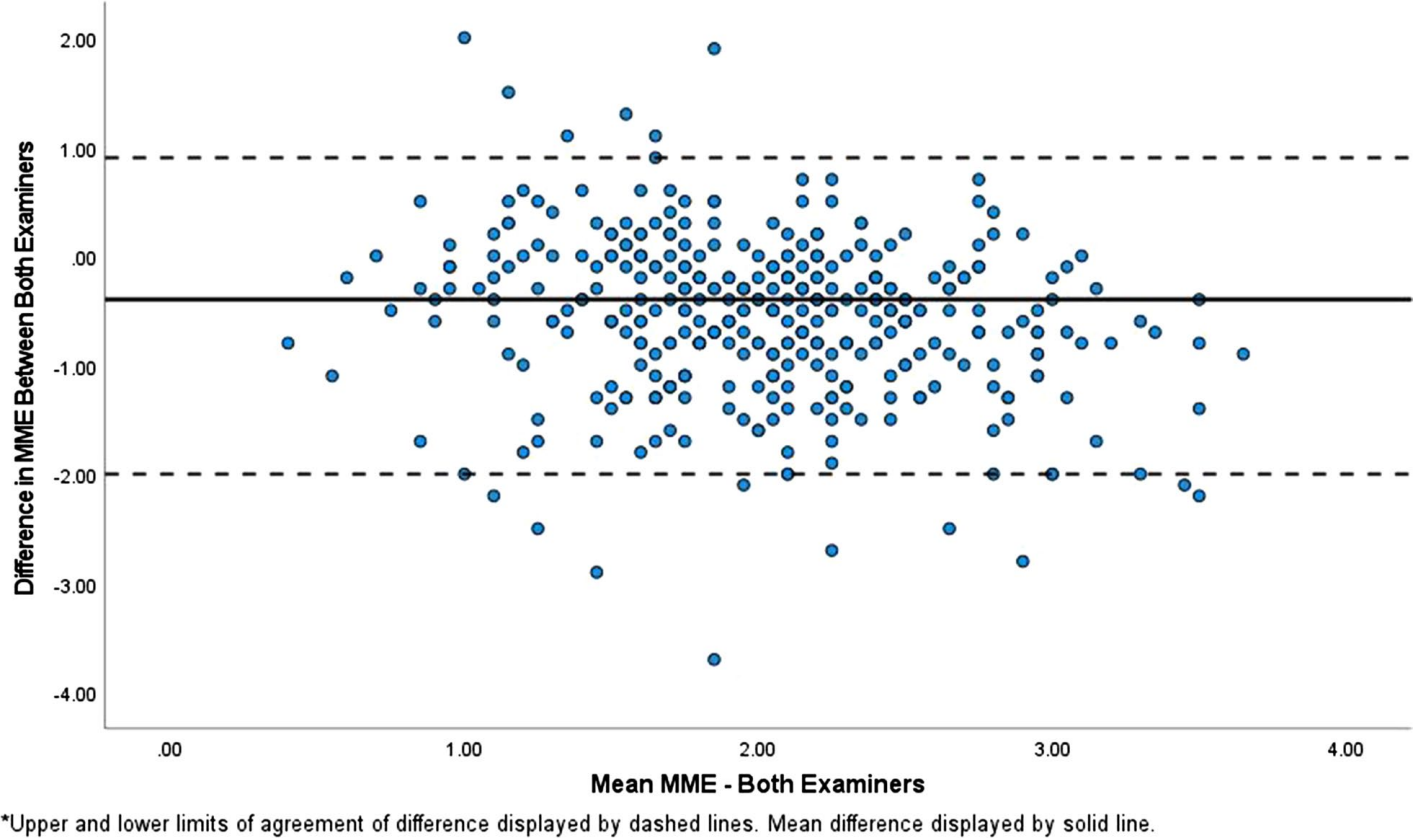
Bland–Altman plot for interexaminer reproducibility. 95% limits of agreement at − 2.0–0.9 mm. The percentage of examiner measurements within 1.0 mm of one another was 75% while the percentage of measurements within 2.0 mm was 97%

Descriptive statistics of MME are reported in Table 1 with the maximum amount of extrusion being with standing (2.8 mm (SD: 0.8); See Fig. 1) and minimum amount with 20° flexion IR (1.9 mm (SD: 0.8); See Fig. 2). The mean and the changes in MME are based on the measurements done by the examiner with more experience in MSK US (JC) because the level of experience (10 years vs 1 year) was deemed appropriate for use as a reference point. Significant change in MME during various positions are reported in Table 2. There was a statistically significant increase in MME in the supine to the standing position, as well as in the 20° flexion IR position to the 20° flexion ER position. Significant decreases in MME were noted between several different positions: (1) standing to 5° IR, (2) standing to 5° ER, (3) 5° IR to 20° IR, (4) 5° ER to 20° IR, (5) standing to 20° ER, and (6) standing to 20° IR. Considering the interexaminer reproducibility, the mean difference between examiner measurements of MME was 0.6 mm (SD: 0.7) with Bland–Altman 95% limits of agreement at − 2.0–0.9 mm (See Fig. 3). The percentage of examiner measurements within 1.0 mm of one another was 75% while the percentage of measurements within 2.0 mm was 97%.

**Table 1:** Mean medial meniscal extrusion (MME) across the six loading phases

Positions	Mean (mm)	Standard deviation (mm)
Supine	2.3	0.5
Standing	2.8*	0.8
5° flexion, internal rotation (IR)	2.3	0.9
5° flexion, external rotation (ER)	2.4	0.7
20° flexion, internal rotation (IR)	1.9**	0.8
20° flexion, external rotation (ER)	2.3	0.7

^*^Maximum amount of extrusion

^**^Minimum amount of extrusion

**Table 2:** Changes in meniscal extrusion during various positions (mm, 95% CI, *p* value)

	Supine	Standing	5° flexion IR	5° flexion ER	20° flexion IR	20° flexion ER
Supine		**0.5** **(0.2–0.7)^**	− 0.05 (− 0.27–0.38)	0.10 (− 0.38–0.18)	− 0.40 (0.05–0.72)	− 0.05 (− 0.27–0.36)
Standing			**0.5** (0.2–0.8)*	**0.4** (0.11–0.65)*	**0.9** (0.53–1.21)*	**0.53** (0.20–0.90)*
5° flexion IR				0.16 (− 0.16–0.48)	**0.33** (0.01–0.65)*	0.01 (− 0.40–0.38)
5° flexion ER					**0.49** (0.19–0.78)*	− 0.15 (− 0.43–0.13)
20° flexion IR						**0.34** **(0.04–0.63)^**
20° flexion ER						

*ER* external rotation, *IR* internal rotation

Bold—Statistically significant

^Statistically significant increase in extrusion

^*^Statistically significant decrease in extrusion

## Discussion

Although the Thessaly test confronts some challenges, we were compelled to investigate direct morphological variation taking place at the medial meniscus under various loading phases of the Thessaly test and to better understand various degrees of MME. The Thessaly test at 20° of flexion showed the highest diagnostic accuracy level of 94% in the diagnosis of medial meniscal tears [9] and our hypothesis was that the greatest degree of MME would be observed at 20° knee flexion.

Our data also showed that the mean MME in healthy normal subjects was 2.3 ± 0.5 mm for the supine and 2.8 ± 0.8 mm for the standing positions. This result conflicts with prior work, where MME of > 3 mm [3, 4] or > 2 mm [5] has shown association with osteoarthrosis, cartilage loss, and medial meniscal tear. Despite prior conclusions of strong associations between MME to various meniscal pathologies, Achtnich and colleagues proposed that the current cut-off value of 3 mm for meniscal pathologies be reconsidered [6]. Although they demonstrated normal physiological degrees of MME, there are some measurement differences compared to our current study (Supine: 2.3 mm ± 0.5 vs. Achtnich et al. 1.1 mm ± 0.5; 20° flexion: 1.9 mm ± 0.8 (IR), 2.3 mm ± 0.7 (ER) vs. Achtnich et al. 1.9 mm ± 0.9). The difference in the measurement is likely due to subtle differences in the measurement reference points. In our study, a line was drawn connecting the free edges of the medial tibial cortex to the medial femoral cortex, whereas in Achtnich and colleagues, a tangential line was drawn on the medial tibia without connecting to the femur. Further, current study includes addition of internal and external rotation to the 20° flexion position which was not considered in prior study by Achtnich. Lastly the difference in measurements may be contributed by the inter/intrarater variability. For instance, Nogueira-Barbosa et al. investigated US assessment of MME using MRI as the reference standard [8]. The study demonstrated moderate and substantial agreement for semiquantitative (ie. grading MME) grading but substantial agreement was achieved when comparing quantitative (absolute values of MME) assessment between the two MSK radiologists (3 years vs 8 years experience). Contrarily, significant increase in the intrarater reliability was found with experience between the PGY-4 resident and experienced physician operator [16].

Noteworthy findings from our study were observed during supine to standing where the largest degree of *increase* in mean MME was noted (refer to Fig. 1), while going from standing to 20° of flexion IR resulted in largest degree of *decrease* (refer to Fig. 2). We found that the mean MME at 20° flexion IR demonstrated the smallest degree of MME; initially, at first glance, least amount of stress applied across the meniscus was assumed, which is contrary to authors’ initial hypothesis. However, upon review of the literature [17–21], a reasonable explanation for this paradoxical reduction of the mean MME at 20° of flexion was found. Both medial and lateral menisci are roughly wedge-shaped in their short axis and with a semilunar longitudinal morphology. It is this wedge-shaped meniscal morphology that converts the vertical load to circumferential tensile loads as the shear forces develop within the menisci, deforming it radially [17]. While the knee undergoes flexion, the menisci conforms to the geometry of the femoral condyle [18]. In flexion, the posterior femoral condyle is in contact with the tibial plateau, which structurally has lesser radii of curvature compared to the contact point of the femoral condyle at extension (i.e. standing), resulting in a decreased contact area [18]. Understanding that the stresses are inversely proportional to the contact area, flexion distributes larger stress across the meniscus. As a result of the different parts of the femoral condyle contacting the tibial plateau during extension and flexion, the medial meniscus withstands posterior displacement while the femoral condyle rolls on the tibial plateau during flexion [18, 21]. The degree of MME could be an indirect measure of assessing the contact area/force relationship applied at the meniscus. These findings reinforce the reason for significant decrease in the mean MME during 20° of flexion which initially was hypothesized to demonstrate the largest degree of MME from greatest applied stress across the meniscus.

Our study demonstrated physiological variations of the medial meniscus during the Thessaly test. Although it does not assume any further diagnostic ability of the Thessaly test, the largest degree of reduction in MME occurred at 20° flexion with IR, which may implicate that this loading phase places most degree of stress among all loading phases. The degree of the MME varies depending on the dynamic phases of the Thessaly test; therefore, the absolute degrees of the extrusion during different loading phases should be investigated in pathologic knees. Future investigation should consider correlating MME variability between age, weight, and gender differences. Further, natural history of physiologically ‘larger’ MME and its progression to osteoarthrosis or tear is still unknown and future investigation is needed.

A secondary objective of our study was to assess whether obtaining measurements of medial meniscal extrusion via ultrasound during the Thessaly test was reproducible. The limits of agreement revealed measurements between examiner 1 and 2 within 2.9 mm (− 2.0–0.9 mm) of one another. Considering 97% of measurements were within 2.0 mm, this leaves only 3% of measurement variability beyond 2.0 mm. To date, other researchers have discovered meniscal pathology suggested by greater than 2.0 mm [4, 5]. Thus, the vast majority of the variability between examiners seems to fall within tolerance for physiological meniscal extrusion.

One of the limitations of the study is the measurement site for the MME. MCL was used as the sole landmark for measurements, thus the posteriorly displaced meniscus could not have been observed during knee flexion. Another limitation is considerable difference in experience between the examiners (10 years vs. 1 year) measuring the MME. While the interexaminer reproducibility assessment was favorable, there was a small negative systematic difference between the examiner measurements. The underlying reason for the underestimation of meniscal extrusion could not be elucidated with certainty but, considering the small difference, probably has little clinical impact.

## Conclusions

Our study demonstrated various degrees of physiological extrusion of the medial meniscus in asymptomatic knees during the loading phases involved in the Thessaly test. Physiological MME does exist and should not be defaulted to pathologic meniscus as previously described. Agreement parameters suggest that measurement of meniscal extrusion during the Thessaly test is reproducible between different examiners.

## Data Availability

The dataset collected and analyzed are available from the corresponding author upon request.

## Abbreviations

IR: Internal rotation
ER: External rotation
MCL: Medial collateral ligament
MME: Medial meniscal extrusion
